# PD-L1 intrinsically promotes the proliferation of breast cancer cells through the SKP2-p27/p21 axis

**DOI:** 10.1186/s12935-024-03354-w

**Published:** 2024-05-09

**Authors:** Marwa Elfoly, Jumanah Y. Mirza, Ayodele Alaiya, Amal A. Al-Hazzani, Asma Tulbah, Monther Al-Alwan, Hazem Ghebeh

**Affiliations:** 1 Cell Therapy and Immunobiology Department, King Faisal Specialist Hospital & Research Centre, Riyadh, 11211 Kingdom of Saudi Arabia; 2 Department of Botany and Microbiology, College of Science, King Saud University, Riyadh, Saudi Arabia; 3 Department of Pathology, King Faisal Specialist Hospital & Research Centre, Riyadh, Saudi Arabia; 4 College of Medicine, Al-Faisal University, Riyadh, Saudi Arabia

**Keywords:** PD-L1, Proliferation, SKP2, p27, p21

## Abstract

**Background:**

PD-L1 intrinsically promotes tumor progression through multiple mechanisms, which potentially leads to resistance to anti-PD-1/PD-L1 therapies. The intrinsic effect of PD-L1 on breast cancer (BC) cell proliferation has not been fully elucidated.

**Methods:**

we used proteomics, gene expression knockdown (KD), quantitative immunofluorescence (qIF), western blots, functional assays including colony-forming assay (CFA) and real-time cell analyzer (RTCA), and in vivo data using immunohistochemistry in breast cancer patients*.*

**Results:**

PD-L1 promoted BC cell proliferation by accelerating cell cycle entry at the G1-to-S phase transition. Global proteomic analysis of the differentially expressed nuclear proteins indicated the involvement of several proliferation-related molecules, including p21^CIP1/WAF1^. Western blotting and qIF demonstrated the higher expression of SKP2 and the lower expression of p21^CIP1/WAF1^ and p27^Kip1^ in PD-L1 expressing (PD-L1^pos^) cells as compared to PD-L1 KD (PD-L1^KD^) cells. Xenograft-derived cells and the TCGA BC dataset confirmed this relationship in vivo. Functionally, CFA and RTCA demonstrated the central role of SKP2 in promoting PD-L1-mediated proliferation. Finally, immunohistochemistry in 74 breast cancer patients confirmed PD-L1 and SKP-p21/p27 axis relationship, as it showed a highly statistically significant correlation between SKP2 and PD-L1 expression (*p* < 0.001), and both correlated significantly with the proliferation marker Ki-67 (*p* < 0.001). On the other hand, there was a statistically significant inverse relationship between PD-L1 and p21^CIP1/WAF1^ expression (*p* = 0.005). Importantly, double negativity for p21^CIP1/WAF1^ and p27^Kip1^ correlated significantly with PD-L1 (*p* < 0.001), SKP2 (*p* = 0.002), and Ki-67 (*p* = 0.002).

**Conclusions:**

we have demonstrated the role of the SKP2-p27/p21 axis in intrinsic PD-L1-enhanced cell cycle progression. Inhibitors of SKP2 expression can alleviate resistance to ICPIs.

**Supplementary Information:**

The online version contains supplementary material available at 10.1186/s12935-024-03354-w.

## Introduction

Breast cancer (BC) is the most commonly diagnosed type of cancer in women [1]. Uncontrolled proliferation is one of the cardinal features of cancer cells. Cells with a higher rate of proliferation eventually form aggressive tumors, leading to disease progression, especially when coupled with features of metastasis [2].

Immune checkpoint inhibitors (ICPIs), pembrolizumab, and atezolizumab are indicated in metastatic BC treatment [3]. ICPIs block immune inhibitory molecules (PD-L1/PD-1) to invigorate the suppressed anticancer immune response [4]. However, PD-L1 has an intrinsic role in promoting tumor progression, independent of its well-established immunomodulatory effects [5]. Unfortunately, ICPIs have limited effect on the intrinsic role of PD-L1, possibly leading to resistance to ICPI therapy. Specifically, In-depth mechanistic studies are needed to understand the underlying mechanisms by which PD-L1 intrinsically affects BC cell proliferation. Hopefully, this will provide a way to combat resistance and improve the overall clinical outcomes of ICPIs/chemotherapy therapies [6].

We and others have identified a key role for intrinsic PD-L1 in promoting BC cell stemness and self-renewal ability [6–8]. While PD-L1 has a clear intrinsic role in maintaining cancer stem cells, we observed significantly larger tumors in PD-L1 expressing (PD-L1^pos^) cells than in their knockdown (KD) counterparts [7]. This was consistent with our previously reported high correlation between PD-L1 and cancer cell proliferation, as measured by Ki-67 and the mitotic index [9]. However, the mechanism of the PD-L1-mediated effect on cancer cell proliferation has not been fully elucidated.

SKP2 is an F-box protein that joins the Skp1-Cullin-F-box (SCF) proteins to form the SCF^SKP2^ complex. SCF^SKP2^ functions as ubiquitin ligase, targeting specific proteins like the cyclin-dependent kinase (CDK) inhibitors p27 and p21 for degradation by the proteasome [10].

Here, we report for the first time that PD-L1 promotes proliferation, at least partially, through regulating the expression of S-Phase Kinase Associated Protein 2 (SKP2), which inhibits the expression of p27^Kip1^ (p27) and p21^CIP1/WAF1^ (p21) to unleash cell cycle progression.

## Materials and methods

### Patient selection

Sections from archived paraffin-embedded Breast cancer samples were obtained from 74 patients diagnosed with invasive ductal carcinoma of the breast and were previously described [9, 11–14]. All patients signed an informed consent approved by KFSH&RC.

### Cell culture and treatment

The MDA-MB-231 cell line (ATCC, USA) and SUM159PT cells (Asterand, USA) were cultured and maintained as previously described [7]. All experiments were performed with mycoplasma-free cells.

Protein expression was measured in the exponential phase (24–48 h) of cell culture as described previously [7], except for p21 or p27, which were more abundant in MDA-MB-231 cells after 30–48 h of culture.

To minimize the effect of the well-established feedback loops in the PI3K/AKT pathway [15], cells were serum-starved for 24-30 h, and the media were exchanged with a complete (serum-supplemented) medium in an 18–24 h culture before analyzing the protein expression of SKP2. We focused on nuclear SKP2, where it interacts with the cell cycle checkpoints p21 and p27.

### Gene expression knockdown

The PD-L1 knockdown (PD-L1^KD^) stable clones, sh-PD-L1(a) and sh-PD-L1(b), and their scrambled shRNA control (Sh-Cont), were previously described [7]. Cells were routinely checked for PD-L1 expression by flow cytometry to confirm their PD-L1 status (Supplementary Fig. 1). PD-L1^KD^ clones of SUM159PT cells were generated as previously described for MDA-MB-231, using the same shRNA vector, and designated as KD1 and KD2, and their scrambled shRNA control Sh-C.

For transient gene KD, cells were transfected with siRNA according to the manufacturer’s instructions using Lipofectamine RNAiMAX transfection reagent (Thermo Fisher Scientific, USA) as previously described [16] (Supplementary Table 1).

### Proteomic analysis

Cytoplasmic and nuclear proteins were fractionated as previously described [17] and were validated using GAPDH and histone H3, respectively, as previously described [7].

Isolated nuclear proteins were further subjected to in-solution tryptic digestion as previously described [18]. Individual proteins were identified by one-dimensional Nano Acquity liquid chromatography coupled with tandem mass spectrometry on Synapt G2 HDMS (Waters, Manchester, UK), as previously described [7]. The data were filtered to show unambiguous protein identification using multiple parameters, including the expected molecular mass.

### Cell cycle analysis

Cells were seeded overnight, washed with PBS, and serum-starved for 24 h before treatment with aphidicolin (4 µg/mL) overnight. Cells were washed twice with PBS, trypsinized, and seeded in complete media. At the indicated time points, cells were harvested, fixed with 70% ethanol, stained with Propidium Iodide, and acquired using a BD FACSCalibur analyzer with BD CellQuest™ Pro software (both from BD Biosciences).

### Colony-forming assay

The cells were seeded in a 6-well plate in complete DMEM at a density of 500 cells/well for 14 days. Colonies were washed with PBS, fixed in 4% formaldehyde, stained with crystal violet and counted manually as previously described [19].

### Xenograft and tissue processing

Xenograft tumors in nude mice previously generated from orthotopic injection of PD-L1^KD^ MDA-MB-231 cells (Sh-PD-L1(a)) or their control (Sh-Cont) [7] were cut into small pieces, digested in a collagenase digestion medium (Stem Cell Technologies, Vancouver, Canada) and agitated at 37 °C for 1 h as previously reported [20]. Single cells were cryopreserved in a 10% DMSO-based freezing medium.

### Cell proliferation assays

#### Manual

Cells (3.5 × 10^4^/T25 flask) were seeded in a complete medium with 4–10% fetal bovine serum (FBS). After 72 h, cells were collected and counted using the trypan blue exclusion dye and a hemocytometer.

#### Automated

The xCELLigence Real-Time Cell Analyzer system (RTCA) (ACEA Biosciences) was used to monitor cell proliferation over time. Cells were seeded at 20,000 cells/well of an E-plate in 200µL of medium. The rate of change in the cell index (CI), which reflects the electronic impedance that directly correlates with cell number, was calculated automatically using RTCA. The data was normalized to the signal recorded 10 h after seeding to disregard unattached/dead cells due to the transfection procedure.

### Western blotting

Proteins were separated by SDS-PAGE and transferred to the PVDF membrane. Membranes were incubated with primary antibodies (Supplementary Table 2) diluted in Tris-buffered saline with Tween20 (TBST) as per the antibody data sheet, followed by the appropriate secondary antibody. The signal was developed using a SuperSignal kit and visualized using an ImageQuant LAS4010 Biomolecular Imager (GE Healthcare, Pittsburgh, PA, USA).

### Quantitative immunofluorescence (qIF)

Protein expression analysis using immunofluorescence (IF) was performed on exponentially growing cells. Cells were cytospinned, air-dried (3–24 h), and immunostained as per Cell Signaling Technology protocol. Primary antibodies for IF are listed in Supplementary Table 2.

A BD pathway 855 Image analyzer and BD AttoVision image acquisition software (BD Biosciences) were used for image capturing and fluorescence quantitation, respectively. Data were analyzed, and the Mean Fluorescence Intensity (MFI) was calculated as previously described [9].

### Immunohistochemistry

Immunohistochemistry was done using formalin-fixed paraffin-embedded (FFPE) tissue sections as previously described [14]. Briefly, tissue sections were dewaxed, rehydrated and blocked for endogenous peroxidase and biotin. Antigen retrieval was done in a Decloaking Chamber pressure cooker (Biocare, Pacheco, USA), as summarized in Supplementary Table 3. The primary antibodies were incubated overnight, while the secondary antibody labeled with horse reddish peroxidase (HRP) was followed by diaminobenzidine (DAB) substrate and the intensity was fortified using CuSO4 (0.6% solution diluted in distilled H_2_O). All washings were done using TBST.

Sections from FFPE blocks of MDA-MB-231 were used as a positive control for SKP2, while MCF-7 was a positive control for p21 and p27 staining.

The anatomical pathologist (AT) scored the expression using 5 to 10 increments. SKP2 and p21 were considered positive if ≥ 10% of infiltrating ductal carcinoma cells had positive nuclear staining, as done previously [21, 22]. p27 was considered positive if ≥ 50% of infiltrating ductal carcinoma cells had positive nuclear staining, as previously established [22]. SKP2 and p27 expression in the tumor-associated immune cells, normal ducts/lobules, and ductal carcinoma in situ (DCIS) was ignored.

Immunohistochemistry of PD-L1 (using SP263 clone of anti-PD-L1 antibody), Ki-67 and the markers of epithelial-to-mesenchymal transition (EMT), vimentin and E-cadherin were previously available and described [9, 13, 14].

### Bioinformatics analysis

Data from publicly available datasets (BC TCGA 2012 nature) were analyzed using the web-based cBioPortal for Cancer Genomics (https://www.cbioportal.org/). Correlation and statistics were provided on the website. In the selected subgroup analysis (i.e., SKP2 high/low), the correlation between PD-L1 and p27 was calculated after data download/extraction, and further analysis and visualization were performed using the publicly available R packages (dplyr and ggplot2).

An in-depth analysis of gene/protein sets obtained from proteomic analysis was performed using ShinyGO, a web-based application (http://bioinformatics.sdstate.edu/go/) for Gene Ontology Enrichment Analysis based on hypergeometric distribution followed by false discovery rate (FDR) correction using a cutoff of 0.1. We used Gene Ontology (GO)-Biological process analysis to link genes with cellular function and GO-Cellular compartment to link genes with different cellular compartments.

### Statistical analysis

All results were normalized to the untreated cells, and data are represented as the mean ± SEM, where replicas are independent experiments. Statistical significance between groups was analyzed using paired Student’s t-test and displayed as * = *p*-value < 0.05, ** = *p*-value < 0.01, *** = *p*-value < 0.001, while # indicates borderline significance.

## Results

### PD-L1 promotes the proliferation of BC cells

We previously observed markedly larger tumors in PD-L1-expressing (PD-L1^Pos^) xenografts than their PD-L1 knockdown (PD-L1^KD^) counterparts (Supplementary Fig. 2) [7]. Whether the PD-L1-mediated increase in tumor size was due to extrinsic factors within the microenvironment or an intrinsic effect of PD-L1 expression on BC cells warranted further investigation. We focused on the intrinsic effect of PD-L1 on cell proliferation as a fundamental tumorigenic feature of cancer cells that can increase tumor size*.*

**Fig. 1:**
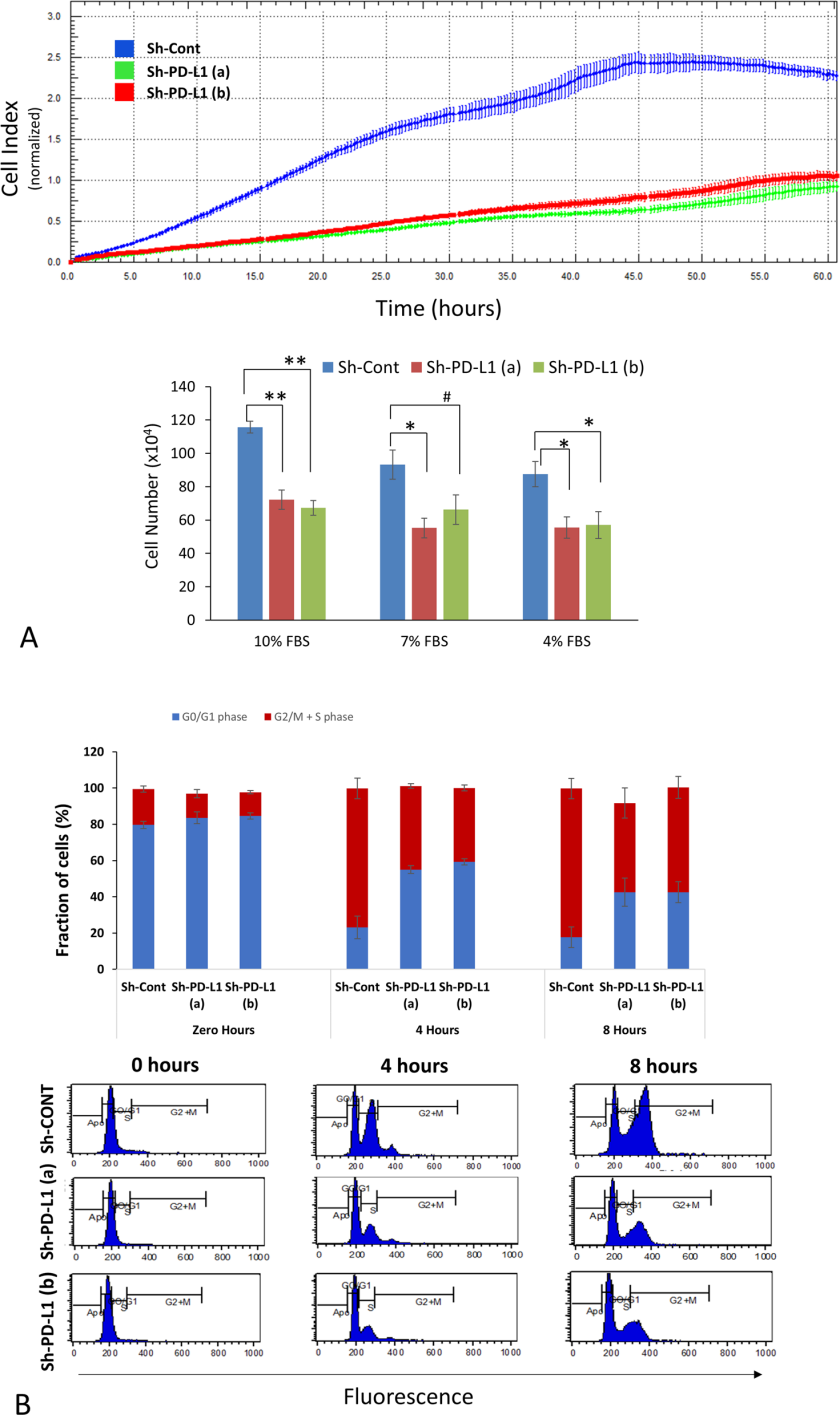
PD-L1 enhances the proliferation and accelerates the cell cycle entry of BC cells. A) Cell proliferation analysis of PD-L1^KD^ MDA-MB-231 clones [Sh-PD-L1 (**a**), Sh-PD-L1 (**b**)] and their control PD-L1^Pos^ (Sh-Cont) using (top) RTCA system (n = 1, cells were cultured in 10% FBS) or (bottom) manual counting (n = 3, cells were cultured in 4–10% FBS) (mean ± SEM). B) Cell cycle analysis using flow cytometry of propidium iodide-stained cells after drug (aphidicolin) washing and complete medium supplementation. Top) Data are presented as the percentage of cells in either G0/G1 or S-G2/M (i.e., cycling) phases and displayed as the mean ± SEM (n = 3). Bottom) Representative flow cytometry cell cycle analysis

PD-L1^Pos^ MDA-MB-231 BC cells proliferated significantly faster than their PD-L1^KD^ clones PD-L1(a) and PD-L1(b), as demonstrated real-time using RTCA in a three-day culture (Fig. 1A, Top). Similarly, a manual assay showed higher proliferation (35–40%) of PD-L1^Pos^ control as compared to PD-L1^KD^ clones (Fig. 1A, bottom). Interestingly, the higher proliferation rate of PD-L1^Pos^ BC cells was maintained even at lower serum concentrations (4 and 7%), suggesting that intrinsic features of PD-L1 are less dependent on growth-promoting factors in serum-supplemented culture medium. We confirmed the effect of PD-L1 expression on promoting proliferation in another BC cell line, SUM159PT (SUM159). PD-L1^Pos^ SUM159 cells (Sh-C) proliferated significantly faster than their KD clones (KD1 and KD2), as shown using RTCA and manual assay (Supplementary Fig. 3).

To further examine the effect of PD-L1 on cell proliferation, we studied its effect on the cell cycle of MDA-MB-231 BC cells using flow cytometry. Random analysis did not reveal major changes in cell cycle phases between PD-L1^KD^ cells and their PD-L1^Pos^ counterparts (data not shown). We hypothesized that the rate of cell cycle entry would be higher in PD-L1^Pos^ cells. To this end, cells were synchronized using aphidicolin (4 µg/mL), a reversible inhibitor that blocks the cell cycle progression at the G1/S entry restriction checkpoint. Within hours of drug washout, PD-L1^Pos^ cells progressed into the other cell cycle phases (S and G2/M) significantly faster than their PD-L1^KD^ counterparts (Fig. 1B). Together, these data support the role of PD-L1 in promoting cancer cell proliferation by accelerating cell cycle entry.

### ***Nuclear proteins of PD-L1***^***Pos***^*** cells hints at the involvement of proliferation-related key molecules***

In order to decipher how PD-L1 promotes cell proliferation, we used a bottom-up approach. We hypothesized that downstream regulators would eventually involve nuclear proteins to transcribe genes related to cell proliferation. This is in addition to the possibility of nuclear nPD-L1 interacting directly with proliferation-related proteins in the nucleus.

We separated the nuclear protein fraction and analyzed differentially expressed proteins (DEP) in PD-L1^Pos^ and PD-L1^KD^ MDA-MB-231 cells using SYNAPT liquid chromatography coupled with tandem mass spectrometry (LC/MS). Among the 897 proteins successfully identified, 451 proteins were DEP with a significant difference (*p* < 0.05) between PD-L1^Pos^ and PD-L1^KD^ cells. Further reduction using a fourfold cut-off yielded 175 DEP between the two groups. Western blotting of a randomly selected protein (EIF1AX) from the proteomic analysis confirmed its downregulation in PD-L1^KD^ clones compared to PD-L1^Pos^ cells (Supplementary Fig. 4).

**Fig. 2:**
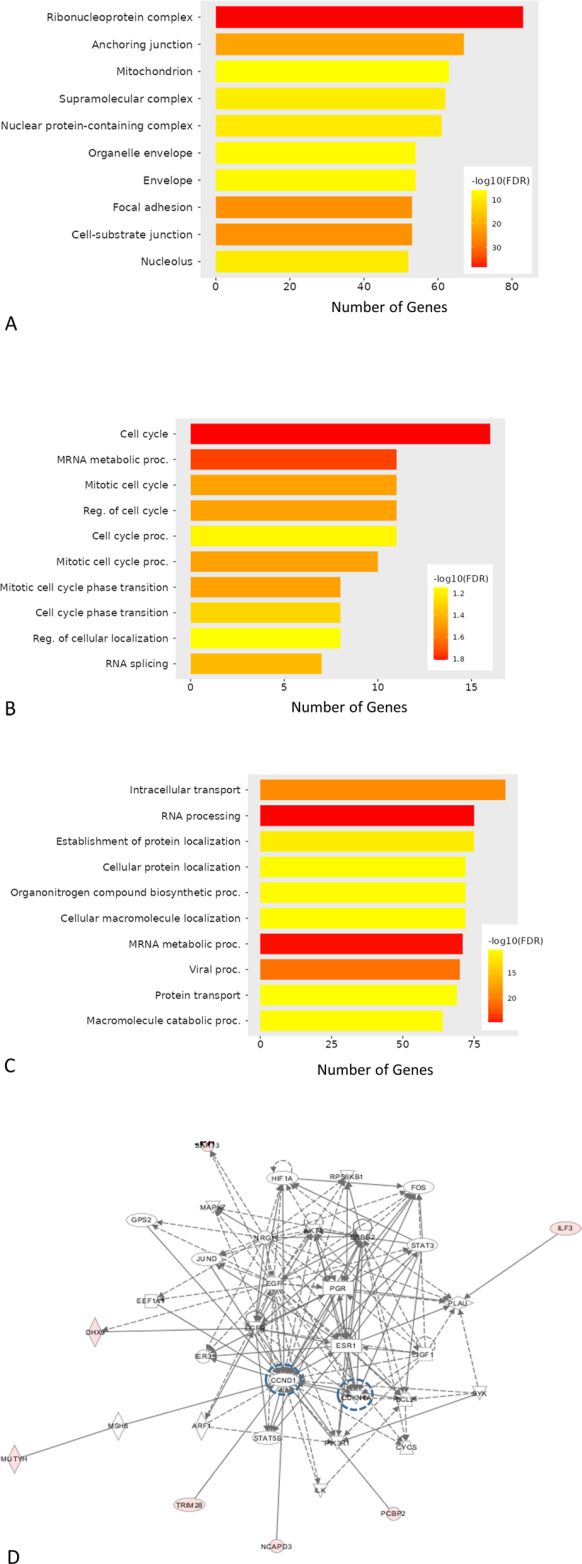
Proteomic analysis of nuclear extracts from PD-L1^Pos^ cells show cell cycle and mitosis-related proteins. Gene/protein enrichment analysis of nuclear extracts using ShinyGo Gene Ontology (GO) cellular component analysis (**A**), GO Biological process analysis of differentially upregulated proteins in PD-L1^Pos^ (**B**) or PD-L1^KD^ cells (**C**). **D** An interaction network is generated based on the DEP using IPA. **E** Pathway analysis of the upregulated proteins using the c-BioPortal genomic website. **F** Protein expression levels (normalized) of the top 25 proteins upregulated in PD-L1^Pos^ control cells

Further analysis using the graphical web-based ShinyGO Gene Enrichment application confirmed that many of the proteomics-identified proteins were de facto nuclear proteins (Fig. 2A). Interestingly, the upregulated proteins were mainly related to cell cycle and mitosis (Fig. 2B). An in-depth literature search showed that out of the 61 overexpressed proteins in PD-L1^Pos^ cells, 41 proteins were previously linked to proliferation, including 19 specifically reported to play a role in the proliferation of BC cells. On the other hand, 390 proteins were downregulated in PD-L1^Pos^ cells compared to their PD-L1^KD^ counterparts, and ShinyGO analysis showed they were mostly related to RNA processing biology (Fig. 2C). Altogether, PD-L1 enriches nuclear proteins that have been reported to regulate the cell cycle and proliferation.

### PD-L1 modulates expression of SKP2-p21/p27 axis

To delineate how these nuclear DEP changes are related to PD-L1-induced proliferation, we deposited them into QIAGEN Ingenuity Pathway Analysis (IPA) software. Multiple networks were suggested, with one having CDKN1A (p21^CIP1/WAF1^) as a central node, in addition to CCND1 (Cyclin D1) (Fig. 2D). Additional IPA analysis of the 175 DEP reduced dataset showed more proliferation key proteins as network hubs, including CDKN2A (p16^Ink4a^), CCND1, HIF-1α (HIF-1), Tp53 (p53), and F-box protein S-phase kinase-associated protein 2 (Skp2) (Supplementary Fig. 5).

**Fig. 3:**
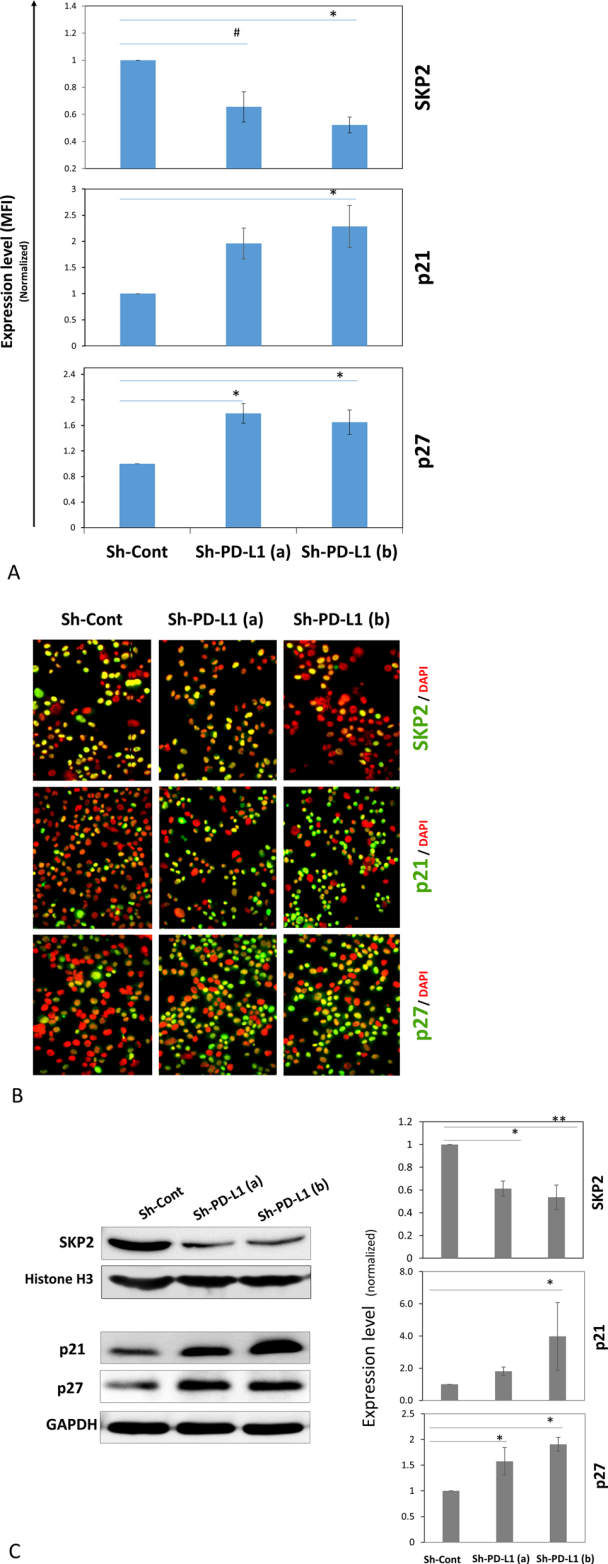
PD-L1 modulates the expression of SKP2, p21 and p27 in BC cells. **A** The expression level of SKP2, p21, p27 in MDA-MB-231 PD-L1^KD^ clones compared with the PD-L1^Pos^ control as measured by qIF. Data were normalized on the MFI of the control and displayed as a mean ± SEM (n = 4). **B** Representative IF images of the PD-L1^KD^ clones and the control (at × 200 magnification). **C** SKP2, p21 and p27 expression following PD-L1^KD^ in MDA-MB-231 cells (left) with quantification of western blots (mean ± SEM, n = 3) (right). Nuclear proteins were used for SKP2, while total protein was used for p21 and p27

There was no significant difference in HIF-1α protein expression, and p16^Ink4a^ was barely detectable in MDA-MB-231 cells (data not shown). p53 is mutated and dysfunctional in MDA-MB-231 cells and thus could not explain the effect on cell proliferation. Cyclin D1 protein expression was unexpectedly increased in PD-L1^KD^ (Supplementary Fig. 6), while SKP2 protein was significantly higher in PD-L1^Pos^ BC cells compared to their PD-L1^KD^ counterparts (Fig. 3A–C). Therefore, we focused on SKP2 as a key proliferation hub.

SKP2 modulates the expression of p27^KIP^ and p21^CIP1/WAF1^ (p27 and p21, respectively) [23], two major checkpoint regulators that block cell cycle progression at the G1/S restriction site. In agreement with the increased expression of SKP2, the expression of p21 and p27 was significantly lower in PD-L1^Pos^ cells than in their PD-L1^KD^ counterparts, as assessed by western blots and qIF (Fig. 3A–C).

To rule out off-target effects of PD-L1 ShRNA, we knocked down PD-L1 transiently using a specific siRNA. In agreement with the results of shRNA PD-L1 KD clones, transient PD-L1^KD^ using siRNA significantly downregulated SKP2 and upregulated p21 and p27 (Supplementary Fig. 7). Interestingly, when SKP2 was KD, p21 and p27 expression increased significantly (Supplementary Fig. 8), confirming SKP2-mediated effect on p21 and p27 expression in MDA-MB-231 cells. The effects of PD-L1 expression on SKP2, p21, and p27 were further tested in SUM159, another BC cell line. In agreement with the results in MDA-MB-231 cells, SKP2 was downregulated, while p21 and p27 were upregulated in PD-L1^KD^ SUM159 cell clones (KD1 and KD2) compared to their PD-L1^Pos^ control (Sh-C) counterparts (Supplementary Fig. 9A–C). Collectively, results confirm the positive effect of PD-L1 on SKP2, which in turn represses p21 and p27 expression.

### PD-L1 promotes proliferation via SKP2

**Fig. 4:**
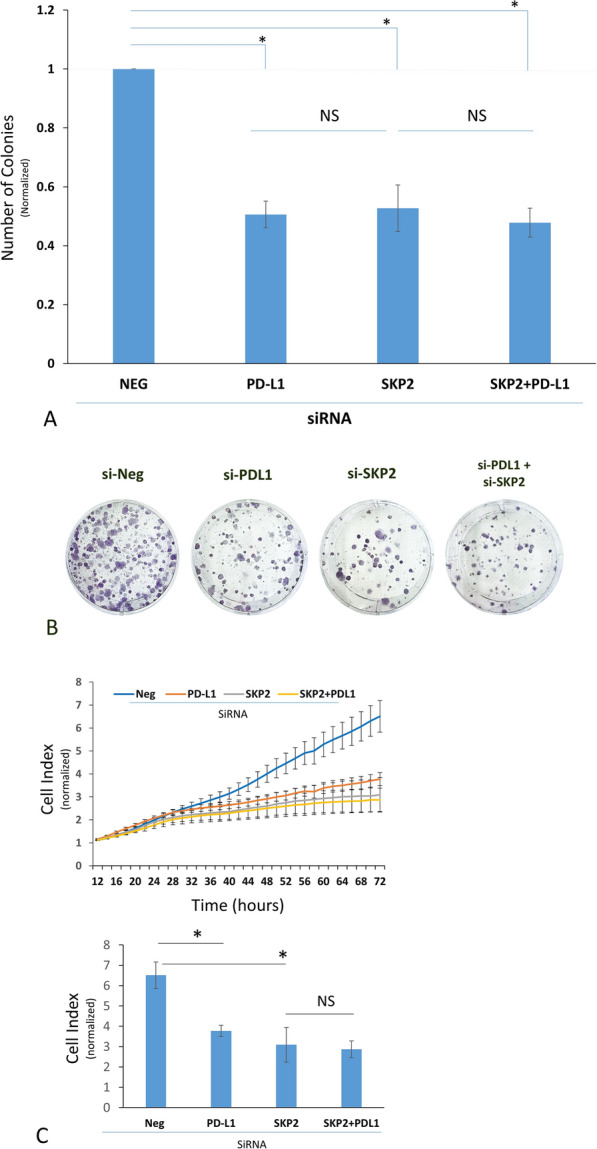
KD of SKP2 expression abrogates the PD-L1-induced colony formation. **A** CFA for MDA-MB-231 cells after transient KD of SKP2 and/or PD-L1. Data were normalized to the colonies formed by the NEG siRNA-treated cells and are displayed as the mean ± SEM (n = 3). **B** Representative CFA upon PD-L and SKP2 KD compared to the control siRNA (**C**) Representative RTCA experiments showing cell proliferation analysis (n = 3) upon KD of PD-L1 ± SKP2 (**A**) compared to the control siRNA. Data (mean ± SEM, n = 3) were normalized to the signal recorded 10 h after seeding to disregard unattached/dead cells due to the transfection procedure

To functionally study the link between PD-L1, SKP2 and cell proliferation, we assessed the CFA for MDA-MB-231 BC cells upon PD-L1 transient KD and its candidate downstream effector, SKP2. PD-L1 and SKP2 KD significantly inhibited the CFA of MDA-MB-231 cells (Figs. 4A and B). PD-L1-mediated effect on CFA was completely abrogated upon SKP2 KD, functionally demonstrating the effect of PD-L1-mediated expression of SKP2 on cell proliferation.

We further tested the effects of PD-L1 and SKP2 KD on the proliferation of MDA-MB-231 cells using RTCA. PD-L1 and SKP2 KD inhibited the proliferation of cells. Significantly, KD of SKP2 (Fig. 4C) abrogated the effect of PD-L1 on cell proliferation. Taken together, our functional data demonstrate that PD-L1 promotes BC proliferation in an SKP2-dependent manner.

### PD-L1 expression correlates with SKP2-p21/p27 in vivo

**Fig. 5:**
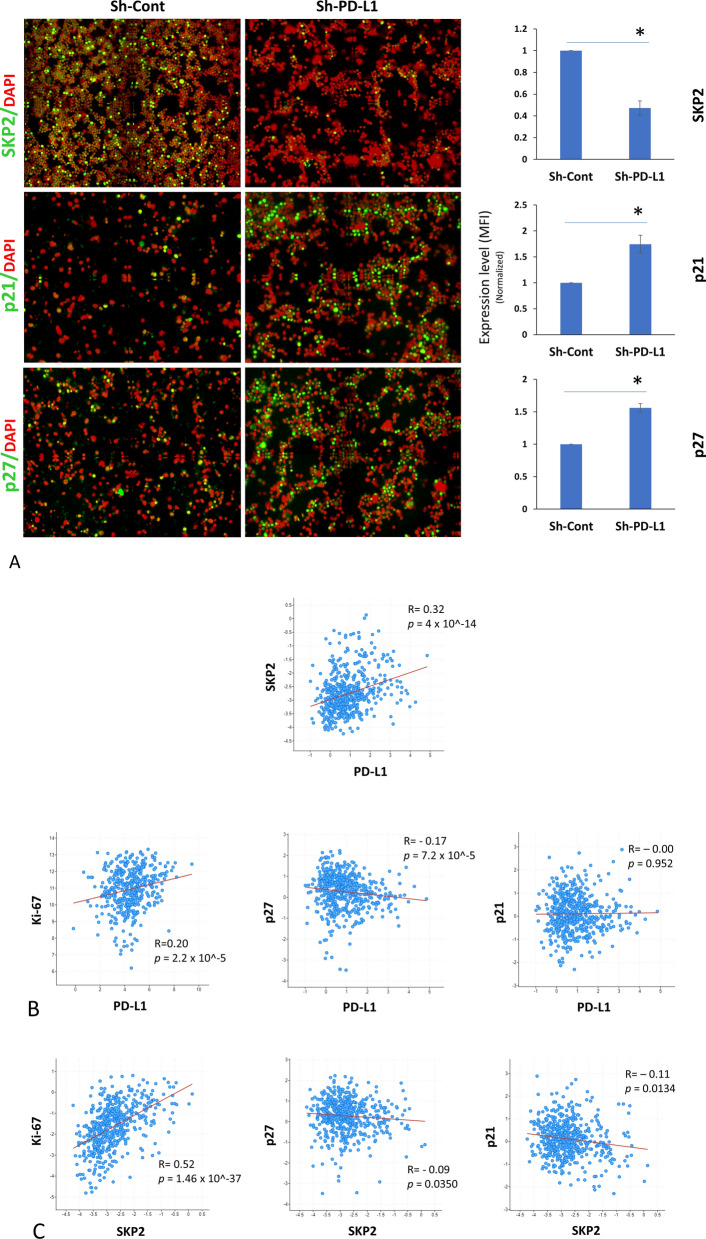

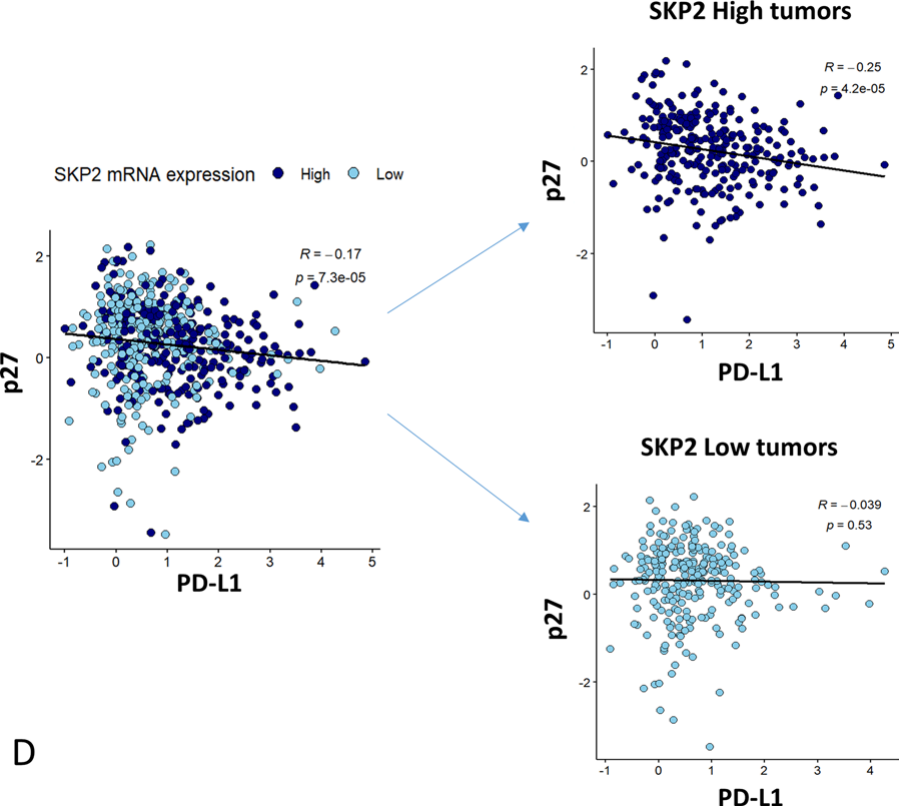
PD-L1 modulates the expression of SKP2, p21, p27 in vivo. Expression of (**A**) SKP2, p21, and p27 in PD-L1^pos^ and PD-L1^KD^ MDA-MB-231 xenografts. Representative IF images (at × 100 magnification) (left) and a bar graph showing the expression level (mean ± SEM, n = 3) as measured by qIF after normalization to the control xenograft cells (Sh-Cont). Correlation between the expression of (**B**) *PD-L1* and *SKP2*, *Ki-67*, *p27*, and *p21*; (**C**) *SKP2* and *Ki-67*, *p27*, and *p21.* Dot plots were generated from the TCGA BC dataset (Nature 2012 mRNA microarray) available in the cBioPortal for Cancer Genomics. **D** The TCGA dataset was further extracted and segregated (based on the median) into *SKP2*^*high*^ and *SKP2*^*low*^, and p27 was plotted against *PD-L1*. The correlation was determined using the Spearman correlation coefficient. *p*-value was calculated using a 2-sample Student t-test for the difference between the two groups of samples

To confirm our findings in vivo, we used cells isolated from cryopreserved enzyme-digested PD-L1^KD^ cells and their control counterparts grown as xenografts in nude mice, as previously reported [7]. In agreement with the in vitro results, SKP2 was downregulated, whereas p27 and p21 were upregulated in xenograft PD-L1^KD^ cells as compared to their PD-L1^Pos^ counterparts (Fig. 5A).

To relate our findings to human patients in vivo data, we examined the relationship between *PD-L1 and SKP2-p21/p27* axis using publicly available datasets. TCGA BC microarray data showed a significant correlation between the expression of *PD-L1* and that of *SKP2* and *Ki-67*, a standard marker of proliferation (Fig. 5B). Furthermore, *PD-L1* showed a significant inverse relationship with *p27* but no significant correlation with *p21* expression. As expected, *SKP2* correlated positively with *Ki-67* and negatively with *p27* and *p21* (Fig. 5C). To test whether the correlation between *PD-L1* and *p27* depends on *SKP2*, we segregated data from the TCGA dataset into *SKP2*^*high*^ and *SKP2*^*low*^ (based on the median). While there was a statistically significant inverse correlation between *p27* and *PD-L1* expression in *SKP2*^*high*^ tumors, no correlation was observed in the *SKP2*^*low*^ group (Fig. 5D). Altogether, the xenograft and TCGA data confirm the relationship between PD-L1 and SKP2-p21/p27 axis in vivo.

### PD-L1 correlates with SKP2-p21/p27 expression in BC patient tissues

While the RNA expression microarray is very useful, it does not always correlate with protein expression in cancer cells, as RNA is not necessarily translated to protein. Therefore, we examined SKP2, p21 and p27 protein expression in BC patients using immunohistochemistry. We used a cohort of 74 patients with previously determined PD-L1 and Ki-67 expression in addition to epithelial-to-mesenchymal transition (EMT) status [9, 13].

**Fig. 6:**
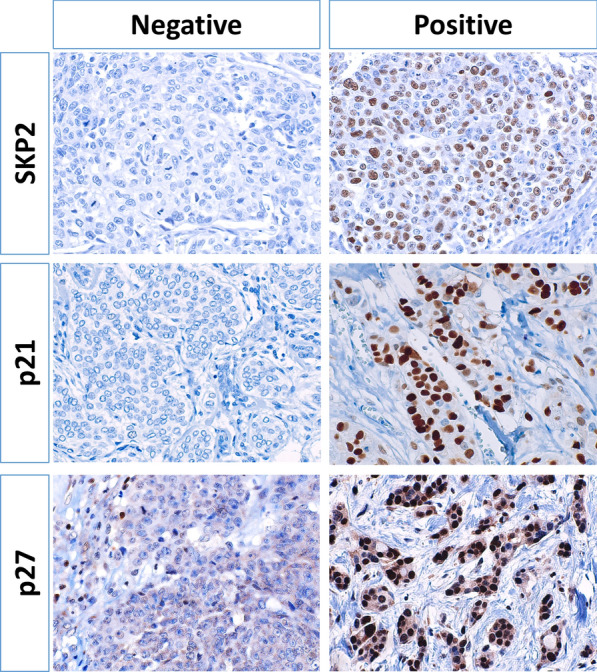
PD-L1 correlates with SKP2, p21/p27 expression in BC patients. Representative images (× 400) for SKP2, p21 and P27 immunohistochemically staining (brown) in BC cancer patients. Hematoxylin is used for counterstaining to show nuclei. Nuclear SKP2, p21 and p27 were scored by anatomical pathologist (AT) using 5 to 10% increments. A positivity cutoff of ≥ 10% was used for SKP2 and p21 and ≥ 50% for p27

p21 (nuclear) was expressed by BC cells in 41% of patients, and its expression correlated inversely with PD-L1 expression (p = 0.005) and SKP2 expression (*p* = 0.013) (Fig. 6 and Table 1). There was no significant correlation between p21 and Ki-67 expression, and p21 expression was mostly restricted to tumor cells.

**Table 1:** Correlation between p21 and p27 expression in tumor cells, with clinicopathological parameters, PD-L1 and SKP2 in 74 breast cancer patients

	p21 (≥ 10%)			p27 (≥ 50%)		*p*
	**−**	**+**	⋄*p*	−	+	
Age						
< 40 years	13 (54)*	11 (46)	0.615	17 (71)	7 (29)	1.00
≥ 40 years	31 (62)	19 (38)		35 (70)	15 (30)	
Tumor size						
< 4 cm	24 (60)	16 (40)	1.00	26 (65)	14 (35)	0.318
≥ 4 cm	20 (59)	14 (41)		26 (76)	8 (24)	
Invasion						
Absent	19 (63)	11 (37)	0.635	18 (60)	12 (40)	0.127
Present	25 (57)	19 (43)		34 (77)	10 (23)	
Histological grade						
1 & 2	22 (59)	15 (41)	1.00	**20 (54)**	**17 (46)**	**0.005**
3	22 (59)	15 (41)		**32 (87)**	**5 (14)**	
Lymph node metastasis^a^						
Absent	17 (68)	8 (32)	0.318	17 (68)	8 (32)	0.789
Present	25 (54)	21 (46)		33 (72)	13 (28)	
ER status						
Negative	20 (69)	9 (31)	0.229	**26 (90)**	**3 (10)**	**0.004**
Positive	24 (53)	21 (47)		**26 (58)**	**19 (42)**	
PR status						
Negative	24 (57)	18 (43)	0.811	**36 (86)**	**6 (14)**	**0.002**
Positive	20 (63)	12 (37)		**16 (50)**	**16 (50)**	
Her2/neu						
Negative	29 (58)	21 (42)	0.803	33 (66)	17 (34)	0.287
Positive	15 (62)	9 (38)		19 (79)	5 (21)	
PD-L1						
< 5%	**31 (52)**	**29 (48)**	**0.005**	32 (63)	19 (37)	0.053
≥ 5%	**13 (93)**	**1 (7)**		20 (87)	3 (13)	
Ki-67						
≤ 20%	23 (55)	19 (45)	0.474	**23 (55)**	**19 (45)**	**< 0.001**
> 20%	21 (66)	11 (34)		**29 (91)**	**3 (9)**	
SKP2						
< 10%	**24 (49)**	**25 (51)**	**0.013**	**30 (61)**	**19 (39)**	**0.030**
≥ 10%	**20 (80)**	**5 (20)**		**22 (88)**	**3 (12)**	
Vimentin						
Negative	27 (53)	24 (47)	0.126	32 (63)	19 (37)	0.053
Positive	17 (74)	6 (26)		20 (87)	3 (13)	
E-Cadherin						
Present	29 (56)	23 (44)	0.438	37 (73)	15 (27)	0.788
Lost	15 (68)	7 (32)		15 (71)	7 (29)	
Vimentin/loss E-Cadherin						
Negative	35 (56)	27 (44)	0.339	42 (68)	20 (32)	0.491
Positive	9 (75)	3 (25)		10 (83)	2 (17)	

^*^(+ and −) are the number of positive and negative patients, The numbers between brackets are the percentages of patients, and **⋄** *p* values in bold and highlighted represent significant data

^a^Three cases with unknown LN involvement

p27 (i.e., nuclear) was expressed in 30% of patients (Fig. 6 and Table 1) with a correlation trend with PD-L1 that did not reach statistical significance. There was a significant inverse correlation between p27 expression in cancer cells and Ki-67 and SKP2 expression (*p* < 0.001 *and p* = 0.030, respectively). Moreover, p27 expression correlated with lower histological grade (p = 0.005), and estrogen and progesterone receptors negativity (*p* = 0.004 and *p* = 0.002, respectively). Interestingly, p27 expression had inverse correlation trend with vimentin expression that did not reach statistical significance.

SKP2 was overexpressed in 34% of BC patients, while it was low or negative in the remaining (Fig. 6 and Table 2). There was a statistical highly significant correlation between SKP2 and PD-L1 expression (*p* < 0.001). Importantly, SKP2 correlated with the expression of the standard proliferation marker, Ki-67 (*p* < 0.001). In addition, SKP2 correlated with high histological grade (p < 0.004), estrogen and progesterone receptors negativity (*p* < 0.001 and *p* = 0.018, respectively). Moreover, SKP2 expression correlated with markers of EMT, vimentin expression (*p* = 0.003) and loss of E-cadherin (*p* = 0.005), and tumors having combined vimentin upregulation and loss of E-cadherin (*p* = 0.002).

**Table 2:** Correlation between SKP2 expression, Lack of both p21 and p27 in tumor cells, with clinicopathological parameters of 74 breast cancer patients

	SKP2 (≥ 10%)		*p	DN p27/p21 (− both, + if either)		*p
	**−**	**+**		**−**	**+**	
Age						
< 40 years	14 (58)	10 (42)	0.432	10 (42)	14 (58)	1.00
≥ 40 years	35 (70)	15 (30)		21 (42)	29 (58)	
Tumor size						
< 4 cm	26 (65)	14 (35)	1.00	15 (38)	25 (62)	0.481
≥ 4 cm	23 (68)	11 (32)		16 (47)	18 (53)	
Invasion						
Absent	19 (63)	11 (37)	0.803	11 (37)	19 (63)	0.482
Present	30 (68)	14 (32)		20 (45)	24 (55)	
Histological grade						
1 & 2	**33 (89)**	**4 (11)**	** < 0.001**	11 (30)	26 (70)	0.059
3	**16 (43)**	**21 (57)**		20 (54)	17 (46)	
Lymph node metastasis^a^						
Absent	15 (60)	10 (40)	0.442	11 (44)	14 (56)	1.00
Present	32 (70)	14 (30)		19 (41)	27 (59)	
ER status						
Negative	**10 (34)**	**19 (66)**	** < 0.001**	**18 (62)**	**11 (38)**	**0.008**
Positive	**39 (87)**	**6 (13)**		**13 (29)**	**32 (71)**	
PR status						
Negative	**22 (52)**	**20 (48)**	**0.006**	20 (48)	22 (52)	0.342
Positive	**27 (84)**	**5 (16)**		11 (34)	21 (66)	
Her2/neu						
Negative	36 (72)	14 (28)	0.189	20 (40)	30 (60)	0.802
Positive	13 (54)	11 (46)		11 (46)	13 (54)	
PD-L1						
< 5%	**47 (78)**	**13 (22)**	** < 0.001**	**19 (32)**	**41 (68)**	** < 0.001**
≥ 5%	**2 (14)**	**12 (86)**		**12 (86)**	**2 (14)**	
Ki-67						
≤ 20%	**37 (88)**	**5 (12)**	** < 0.001**	**11 (26)**	**31 (74)**	**0.002**
> 20%	**12 (38)**	**20 (62)**		**20 (63)**	**12 (37)**	
Vimentin						
Negative	**42 (82)**	**9 (18)**	** < 0.001**	**16 (31)**	**35 (69)**	**0.010**
Positive	**7 (30)**	**16 (70)**		**15 (65)**	**8 (35)**	
E-Cadherin						
Present	**41 (79)**	**11 (21)**	** < 0.001**	22 (42)	30 (58)	1.00
Lost	**8 (36)**	**14 (64)**		9 (41)	13 (59)	
Vimentin & loss E-Cadherin						
Negative	**47 (76)**	**15 (24)**	** < 0.001**	24 (38)	38 (62)	0.223
Positive	**2 (17)**	**10 (83)**		7 (58)	5 (42)	

*DN* Double negative

^*^(+ and −) are the number of positive and negative patients, the numbers between brackets are the percentages of patients, and **⋄** *p* values in bold and highlighted represent significant data

^a^Three cases with unknown LN involvement

While SKP2 regulates both p21 and p27, other mechanisms can regulate each cell cycle checkpoint individually. Therefore, we looked at tumors that are negative for both p21 and p27. Tumors lacking both p21 and p27 (double negative, DN) represented 58% of patients. Indeed, DN tumors correlated with SKP2 expression (*p* = 0.003). Importantly, DN tumors correlated with PD-L1 expression (*p* = 0.001) and Ki-67 positivity (*p* = 0.006) (Table 2). In addition, DN tumors correlated with high histological grade (*p* = 0.049), estrogen receptor negativity (p = 0.004), and vimentin expression positivity (*p* = 0.009).

In summary, our data demonstrate the intrinsic effect of PD-L1 on upregulating SKP2 expression, leading to the downregulation of p27 and p21 expression to unleash cell cycle progression and promote cell proliferation.

## Discussion

Accumulating evidence supports the intrinsic effects of PD-L1 on cancer cells, in addition to its established role as an immune checkpoint. We and others have reported a strong correlation between PD-L1 expression and the proliferation markers (Ki-67) and mitotic index [9, 24–26] in BC. Despite this strong association, the mechanism underlying PD-L1-induced cell proliferation in BC is still not well-defined. In this study, we have demonstrated for the first time that PD-L1 promotes BC cell proliferation by modulating the SKP2-p27/p21 axis.

The intrinsic role of PD-L1 in promoting BC cell proliferation reported in this study is consistent with previous results for BC [27, 28] and other types of cancer [29, 30]. Interestingly, the effect of PD-L1 on cell proliferation was maintained at reduced (4–7%) serum concentrations, suggesting the observed effect was likely mediated by constitutively activated intracellular signaling pathways rather than extracellular signals like growth factors. BC cells commonly harbor PIK3CA, AKT1, and HRAS/KRAS mutations, as well as PTEN deletion/mutations [31], leading to the constitutive activation of proliferation-related pathways such as the PI3K/AKT and MAPK/ERK pathways. Indeed, we and others have previously shown the intrinsic effect of PD-L1 in BC involves mainly the PI3K/AKT pathway and the ERK/MAPK pathway [6, 7, 32].

The PI3K/AKT pathway promotes cell cycle progression through multiple mechanisms that do not necessarily coexist in one type of cancer or under different conditions. For example, while the PI3K/AKT pathway can promote Cyclin D1 expression, this was not the case in our MDA-MB-231 BC cells (Supplementary Fig. 6). Our results have demonstrated for the first time that intrinsic PD-L1 can upregulate SKP2 and downregulate p27 and p21 expression to promote BC proliferation.

SKP2 overexpression in BC is directly linked to the induction of proliferation and other oncogenic features [33]. SKP2 protein levels are regulated by gene expression and protein stability, which are heavily regulated by the PI3K/AKT pathway [34]. The PD-L1 effect on SKP2 is likely to be indirect through posttranslational modification as it was previously shown that AKT regulates SKP2 expression by its effect on SKP2 Ser72 phosphorylation [35].

Expression of *PD-L1* in the microarray-based BC TCGA public dataset showed a highly significant negative correlation with *p27* but not with *p21*. It is possible that PD-L1 regulates p21 protein expression at the post-translational level since PI3K/AKT and SKP2 were reported to regulate the protein level of p21 through both transcriptional and non-transcriptional mechanisms [36–38].

RNA expression microarray is very useful due to the publically available datasets, which have a relatively large number of samples. However, RNA expression data do not always correlate with protein expression, as RNA is not necessarily translated to protein. Importantly, cancer cells are not the only source of mRNA in the tissue, which includes stromal cells composed of immune, endothelial and mesenchymal cells.. Notably, a large portion of some BC tissues are actually ductal carcinoma in situ (DCIS) and, therefore, are not representative of the invasive ductal carcinoma component of BC. Indeed, using immunohistochemistry, we have demonstrated a statistically significant inverse correlation between PD-L1 and p21 expression on the protein level.

We have shown for the first time that PD-L1 correlates with SKP2 overexpression in BC. Importantly, we have demonstrated functionally that PD-L1 promotes proliferation by modulating SKP2 expression. Moreover, in the cohort of tested BC patients, SKP2 correlated with high histological grade, Ki-67 expression, estrogen receptor and progesterone receptor negativity, which is in agreement with previous reports [22, 39]. Similarly, SKP2 correlated significantly with vimentin upregulation (*p* < 0.001), loss of E-cadherin (*p* = 0.005) and tumors having both vimentin upregulation and loss of E-cadherin (*p* = 0.005), which is consistent with previously described reports [40]. The correlation of SKP2 expression with markers of EMT demonstrates similarity with PD-L1 expression, which also correlates with EMT [13].

There was no significant correlation between p21 and Ki-67 in the study, which is consistent with previous studies [21, 41, 42].

Nuclear p27 is often decreased in BC and other human cancers in general, which is associated with poor prognosis. The degradation of p27 in cancer is usually mediated by SKP2-mediated proteolysis in addition to mRNA expression-based mechanisms, including miRNA. Interestingly, in the current work, the expression of p27 correlated inversely with estrogen and progesterone receptor negativity (*p* < 0.001) and the proliferation marker Ki-67 positivity (*p* = 0.004), which is consistent with previous reports [22]. The lack of significant correlation between p27 and PD-L1 or SKP2 in immunohistochemistry could be due to low sample size (as the trend was there) or the fact that p27 is regulated by both SKP2-dependent and SKP2-independent mechanisms [43]. Since p21 and p27 are substrates for SKP2, assessing tumors that are double negative (DN) for both p27 and p21 would more likely correlate with SKP2 expression. Indeed, p21/p27 DN tumors correlated with SKP2 positive, Ki-67 positive and PD-L1 positive status.

In conclusion, our findings using different BC cell lines in vitro in addition to in vivo xenografts and patient samples clearly demonstrated the intrinsic effect of PD-L1 on the SKP2-p21/p27 axis and the consequence of this relationship on cell proliferation. These findings expand our understanding of PD-L1 intrinsic function beyond its well-established immunomodulatory effects and identify the underlying effectors, which could be targeted in future combination therapy.

### Supplementary Information

**Supplementary file 1. Supplementary Figure 1.** shRNA-mediated PD-L1 knockdown in clones (a & b) of MDA-MB-231 breast cancer cells was routinely confirmed by flow cytometry. MFI=Mean Fluorescence Intensity.

**Supplementary file 2. Supplementary Figure 2.** Total xenograft tumor volume of all tumors formed from PD-L1^KD^ cells (Sh-PD-L1(a)) as compared to PD-L1^Pos^ control (Sh-Cont) calculated as Tumor volume = ½ (Length*Width^2^). Data were collected from previously described mice experiments [7].

**Supplementary file 3. Supplementary Figure 3.** Cell proliferation (mean ± SEM) of PD-L1^KD^ SUM159 clones (KD1 and KD2) and their control (Sh-C) using (top) RTCA system (n=1) or (bottom) manual counting.

**Supplementary file 4. Supplementary Figure 4.** Expression of EIF1AX in the PD-L1^KD^ clones Sh-PD-L1(a) and Sh-PD-L1(b) of MDA-MB-231 cells compared with the control (Sh-Cont) measured by western blot (left) with quantification of blots (mean ± SEM, n=3) (right).

**Supplementary file 5. Supplementary Figure 5.** Interaction network generated using IPA based on the differentially modified nuclear proteins from proteomic with a 4-fold difference between PD-L1^KD^ clones (PD-L1(a) and PD-L1(b)) and their PD-L1^Pos^ control (Sh-Cont).

**Supplementary file 6. Supplementary Figure 6.** A) qIF showing the expression of Cyclin D1 in the PD-L1^Pos^ control MDA-MB-231 cells (Sh-Cont) and PD-L1^KD^ clones ShPD-L1(a) and Sh-PD-L1(b). Data were mean ± SEM (n=3) after normalization on the expression level of Sh-Cont. B) Representative IF images of the PD-L1^KD^ clones and Sh-Cont (at x100 magnification).

**Supplementary file 7. Supplementary Figure 7.** Left) The expression level of SKP2, p21, p27 in MDA-MB-231 cells upon transient KD of PD-L1 using specific siRNA or scrambled siRNA (siNeg) as a control. qIF showing protein expression (MFI) after normalization on siNEG cells. Data are displayed as the mean ± SEM (n=4). Right) Representative IF images upon the transient KD or the si-Neg (at x200 magnification).

**Supplementary file 8. Supplementary Figure 8.** A) The expression level of p21 and p27 in MDA-MB-231 upon transient KD of SKP2 using si-SKP2 or scrambled siRNA (si-Neg). (Right) MFI was measured by qIF after normalization on the control (si-Neg) and displayed as a mean±SEM (n=4). (Left) Representative IF images upon transient KD using siNEG or si-SKP2 (at x200 magnification).

**Supplementary file 9. Supplementary Figure 9.** A) qIF showing the MFI of SKP2, p21, p27 in SUM159 PD-L1^KD^ clones KD1 and KD2 after normalization on the control (Sh-C) (n=1). B) Representative IF images of the PD-L1^KD^ clones and the control (at x200 magnification). C) Western blot showing SKP2 and p27 expression following PD-L1^KD^ in SUM159 cells (left) with quantification of blots (mean±SEM, n=3) (right) for SKP2 and n=1 for p27.

**Supplementary file 10. Supplementary Table 1.** SiRNA sequence.

**Supplementary file 11. Supplementary Table 2.** List of Antibodies used.

**Supplementary file 12. Supplementary Table 3.** Immunohistochemistry conditions.

s

## Data Availability

The data generated in this current study are included in this published article (and its supplementary information files), otherwise available from the corresponding author on reasonable request.

## Abbreviations

PD-L1: Programmed death ligand-1
DEP: Differentially expressed proteins
Skp2: S-phase kinase-associated protein 2
DCIS: Ductal Carcinoma in Situ
