# Supplementary figures and images for: PD-L1 intrinsically promotes the proliferation of breast cancer cells through the SKP2-p27/p21 axis
Source: Cancer Cell Int. 2024 May 9;24:161. doi: 10.1186/s12935-024-03354-w (PMC11084005; doi:10.1186/s12935-024-03354-w)

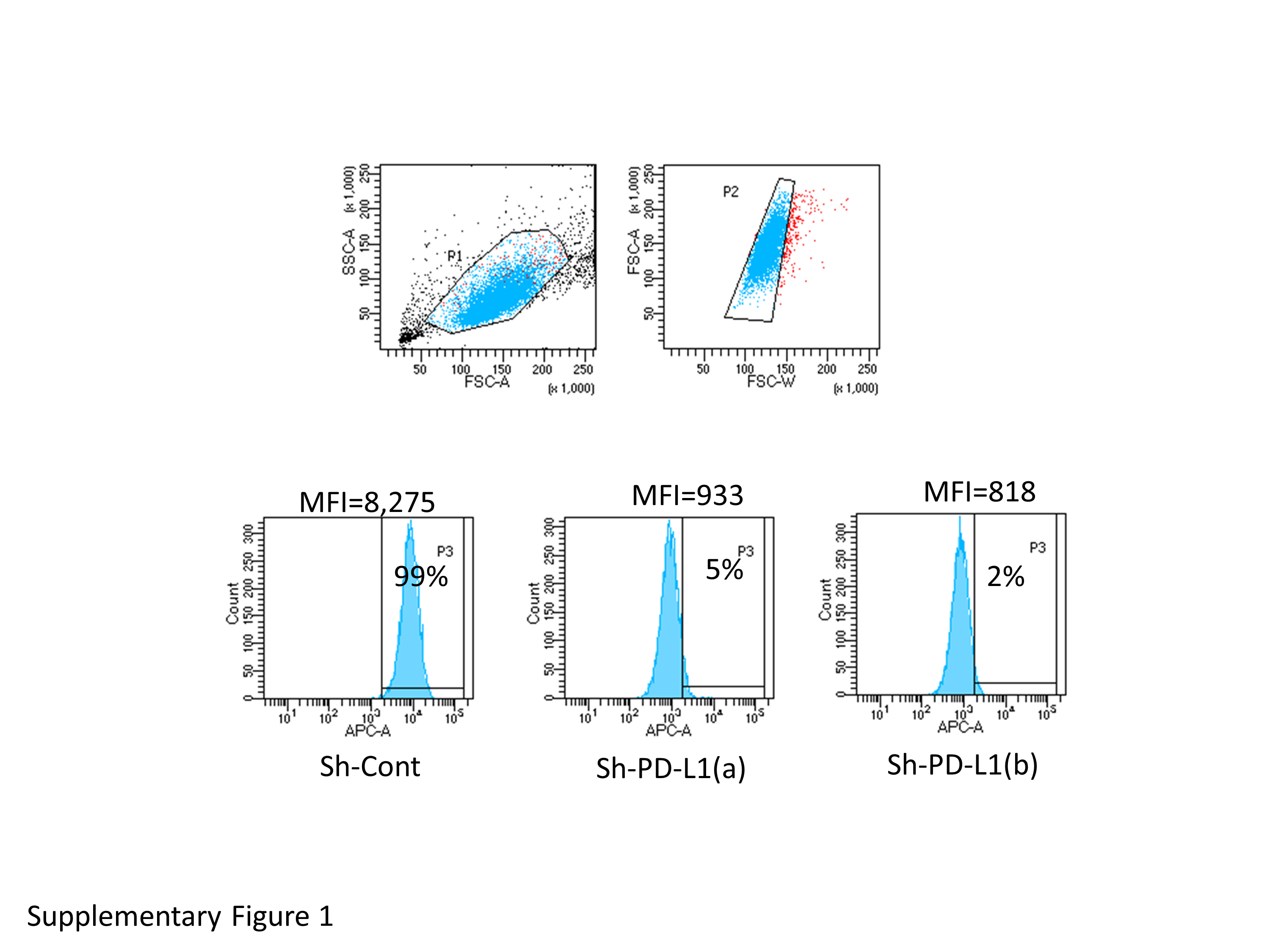

Supplement: Supplementary file 1 — Supplementary Figure 1. shRNA-mediated PD-L1 knockdown in clones (a & b) of MDA-MB-231 breast cancer cells was routinely confirmed by flow cytometry. MFI=Mean Fluorescence Intensity. [file 12935_2024_3354_MOESM1_ESM.tif]

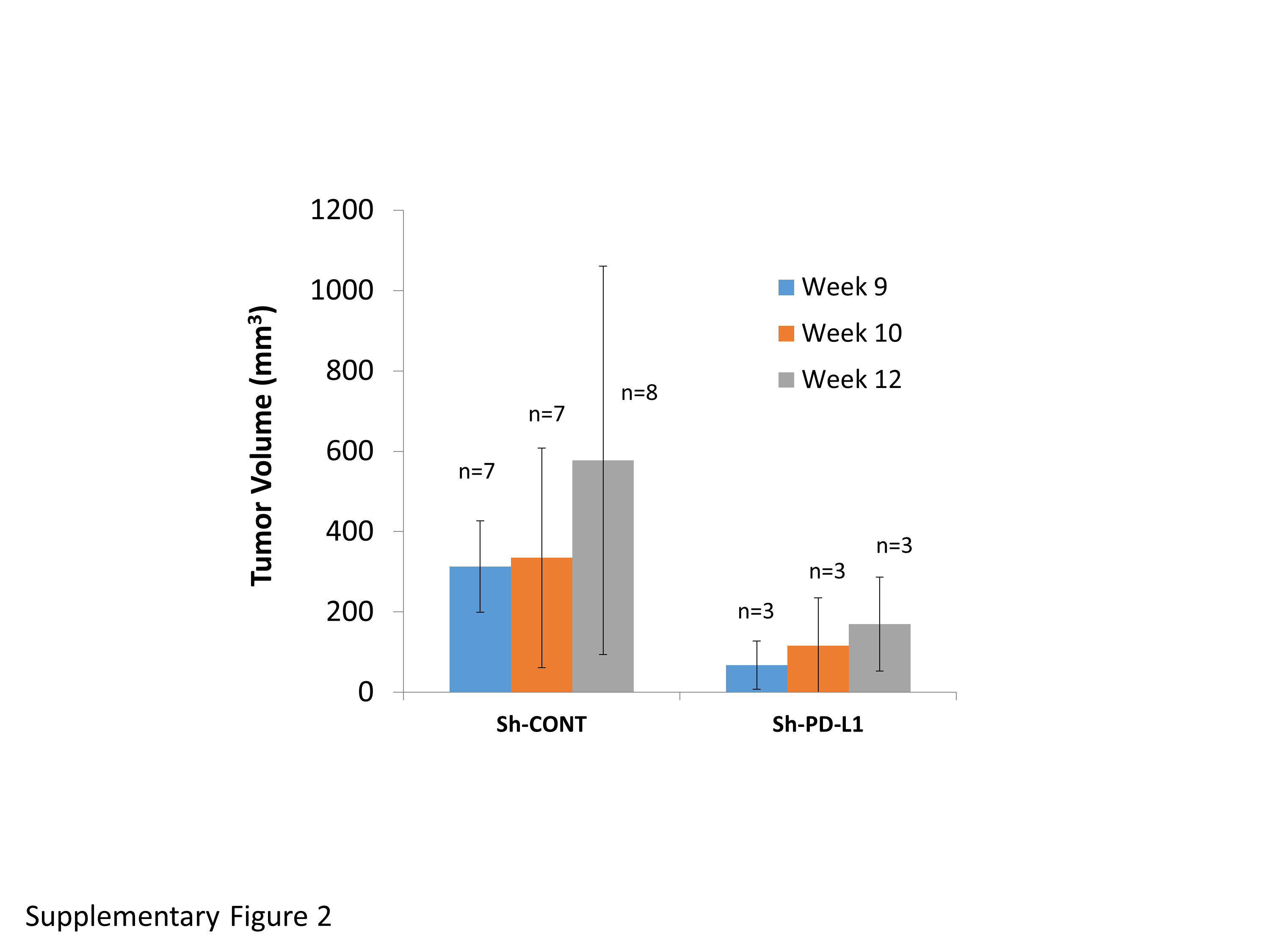

Supplement: Supplementary file 2 — Supplementary Figure 2. Total xenograft tumor volume of all tumors formed from PD-L1KD cells (Sh-PD-L1(a)) as compared to PD-L1Pos control (Sh-Cont) calculated as Tumor volume = ½ (Length*Width2). Data were collected from previously described mice experiments [7]. [file 12935_2024_3354_MOESM2_ESM.tif]

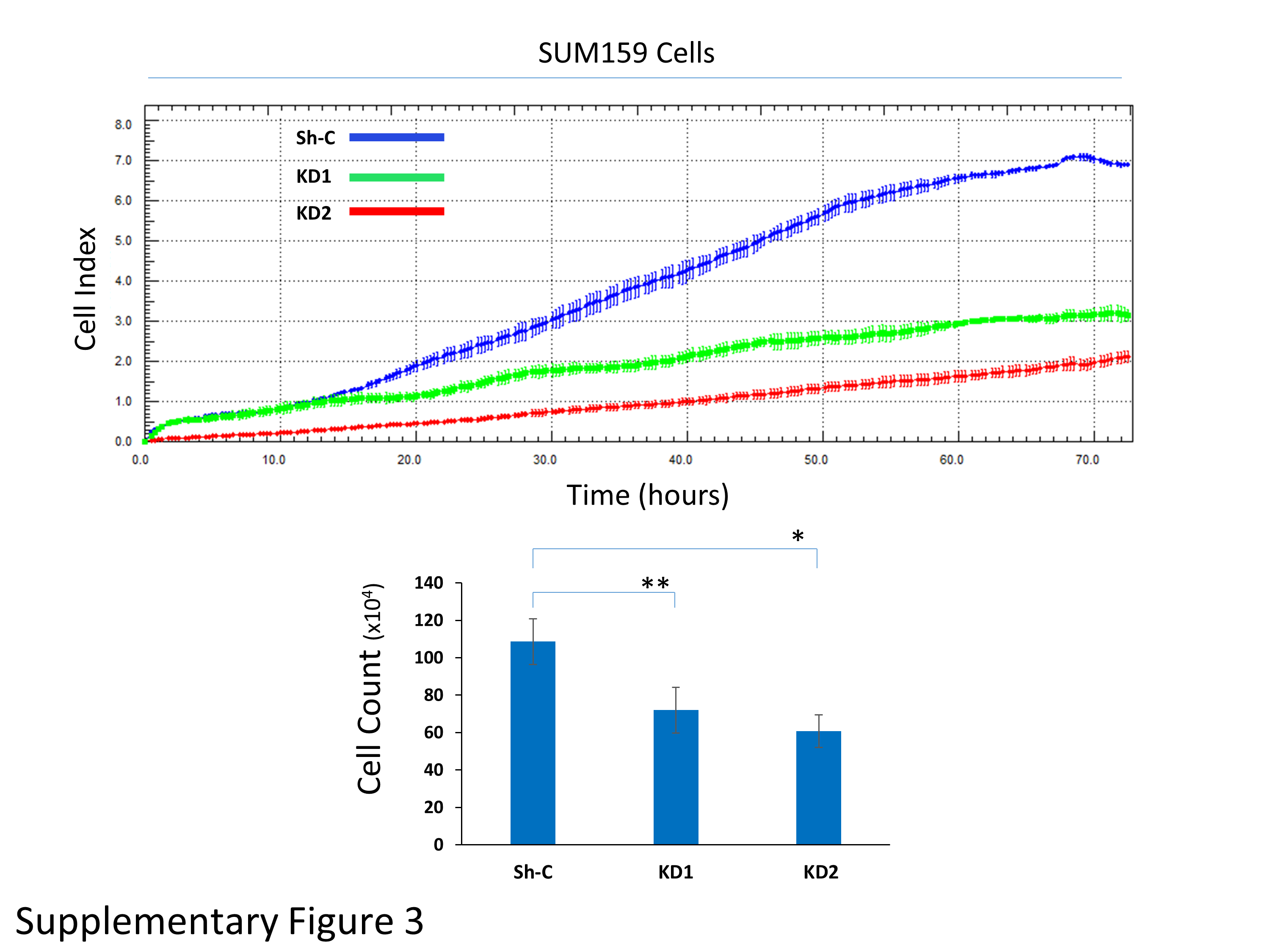

Supplement: Supplementary file 3 — Supplementary Figure 3. Cell proliferation (mean ± SEM) of PD-L1KD SUM159 clones (KD1 and KD2) and their control (Sh-C) using (top) RTCA system (n=1) or (bottom) manual counting. [file 12935_2024_3354_MOESM3_ESM.tif]

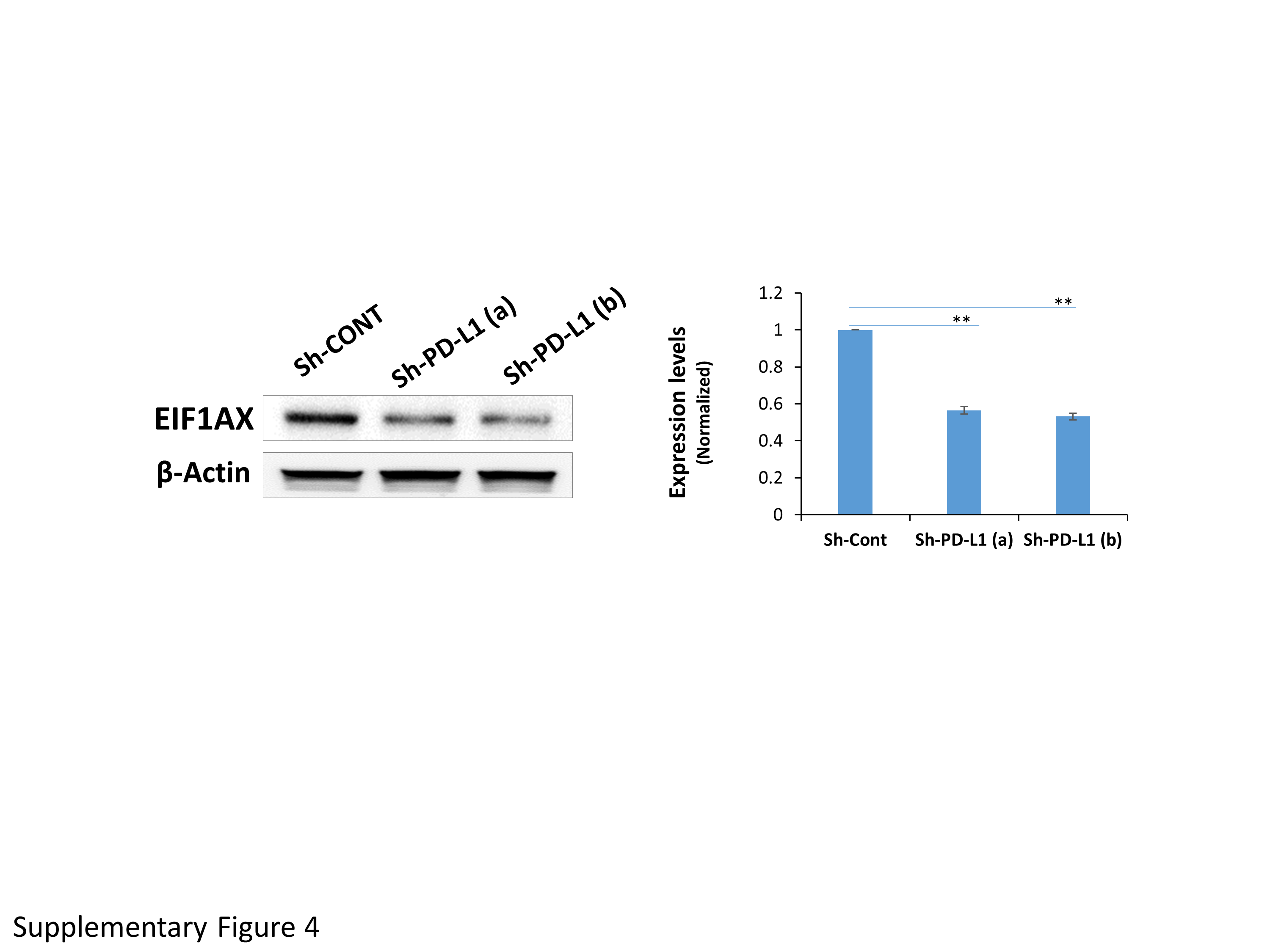

Supplement: Supplementary file 4 — Supplementary Figure 4. Expression of EIF1AX in the PD-L1KD clones Sh-PD-L1(a) and Sh-PD-L1(b) of MDA-MB-231 cells compared with the control (Sh-Cont) measured by western blot (left) with quantification of blots (mean ± SEM, n=3) (right). [file 12935_2024_3354_MOESM4_ESM.tif]

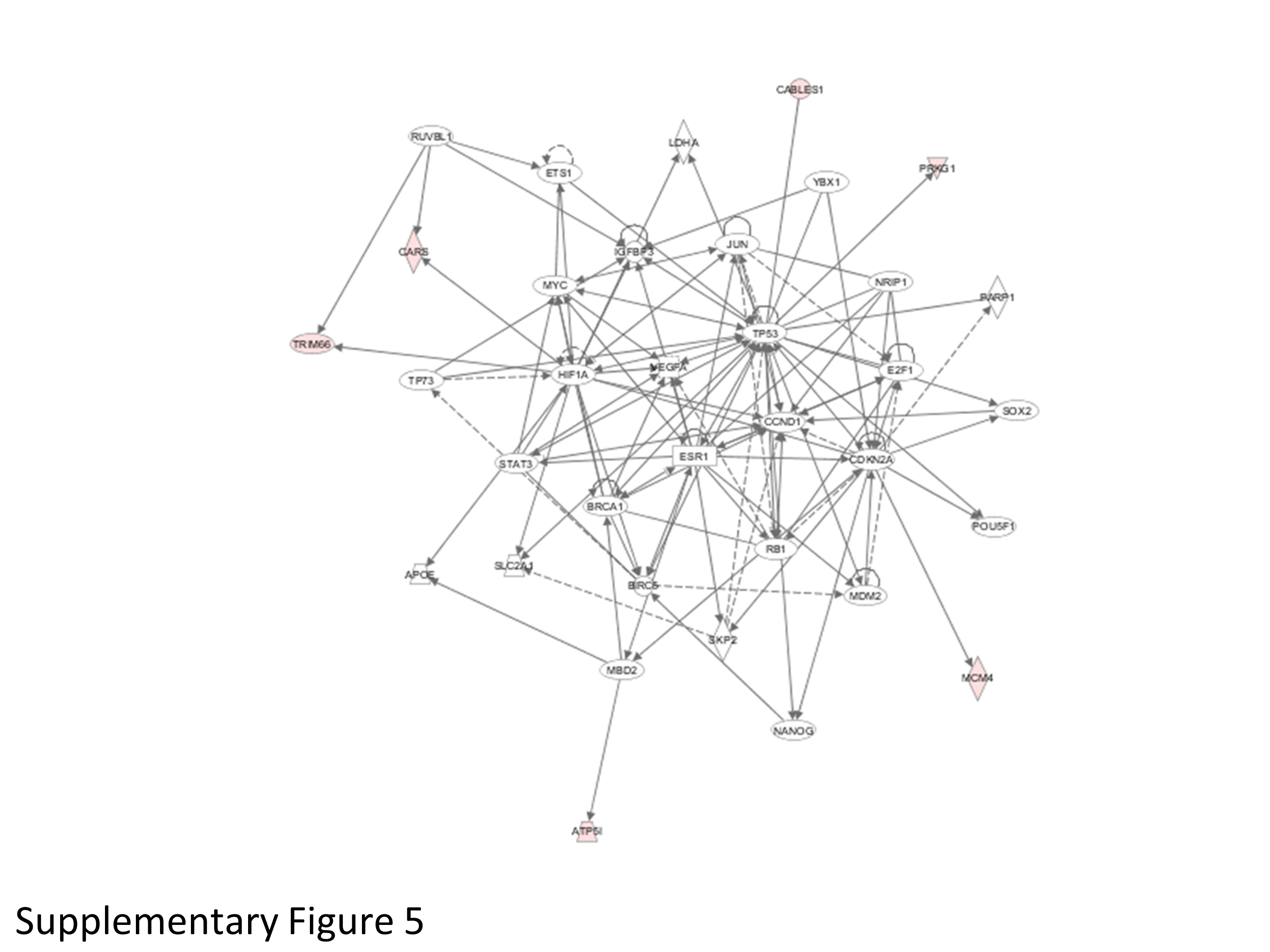

Supplement: Supplementary file 5 — Supplementary Figure 5. Interaction network generated using IPA based on the differentially modified nuclear proteins from proteomic with a 4-fold difference between PD-L1KD clones (PD-L1(a) and PD-L1(b)) and their PD-L1Pos control (Sh-Cont). [file 12935_2024_3354_MOESM5_ESM.tif]

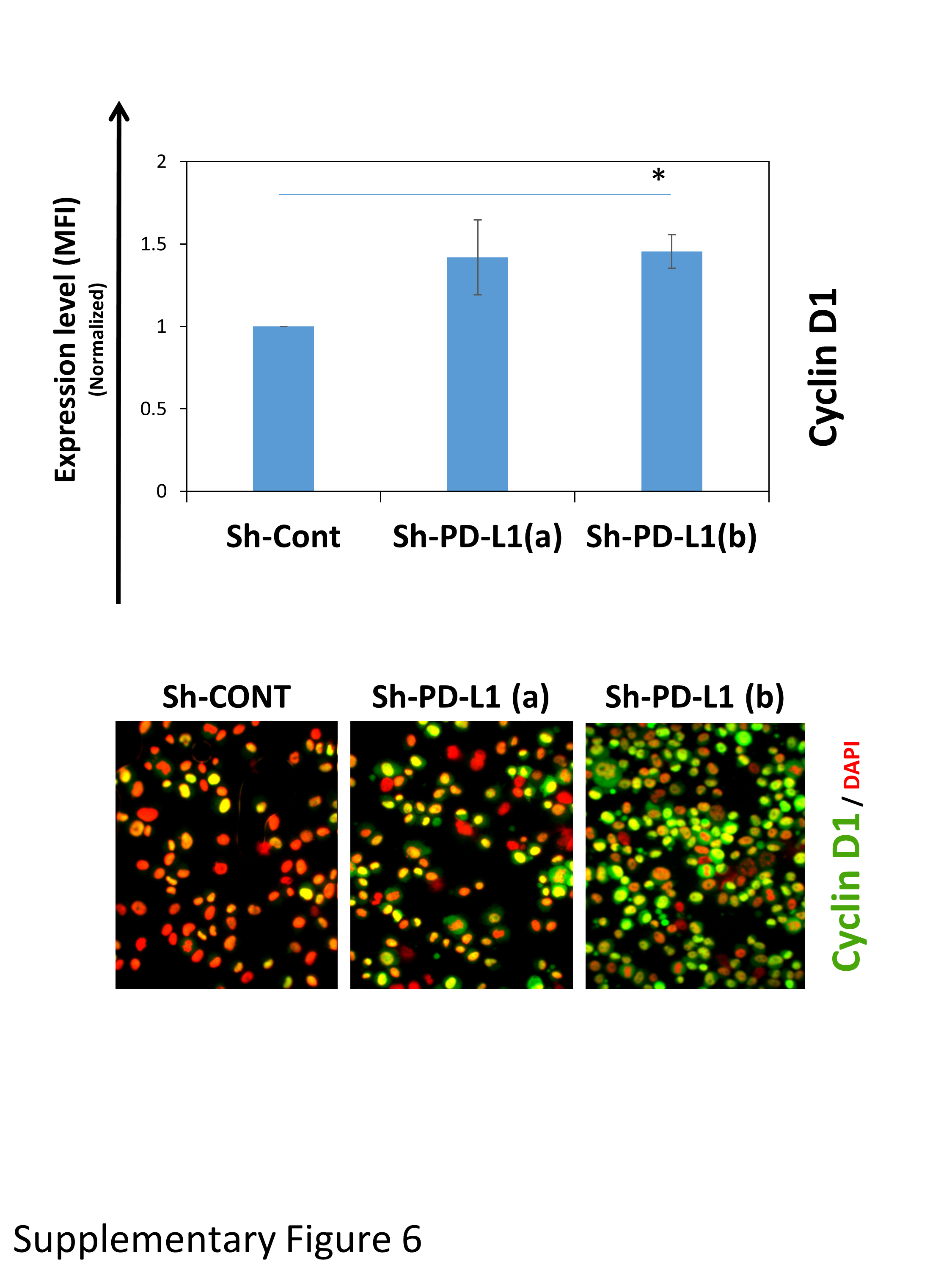

Supplement: Supplementary file 6 — Supplementary Figure 6. A) qIF showing the expression of Cyclin D1 in the PD-L1Pos control MDA-MB-231 cells (Sh-Cont) and PD-L1KD clones ShPD-L1(a) and Sh-PD-L1(b). Data were mean ± SEM (n=3) after normalization on the expression level of Sh-Cont. B) Representative IF images of the PD-L1KD clones and Sh-Cont (at x100 magnification). [file 12935_2024_3354_MOESM6_ESM.tif]

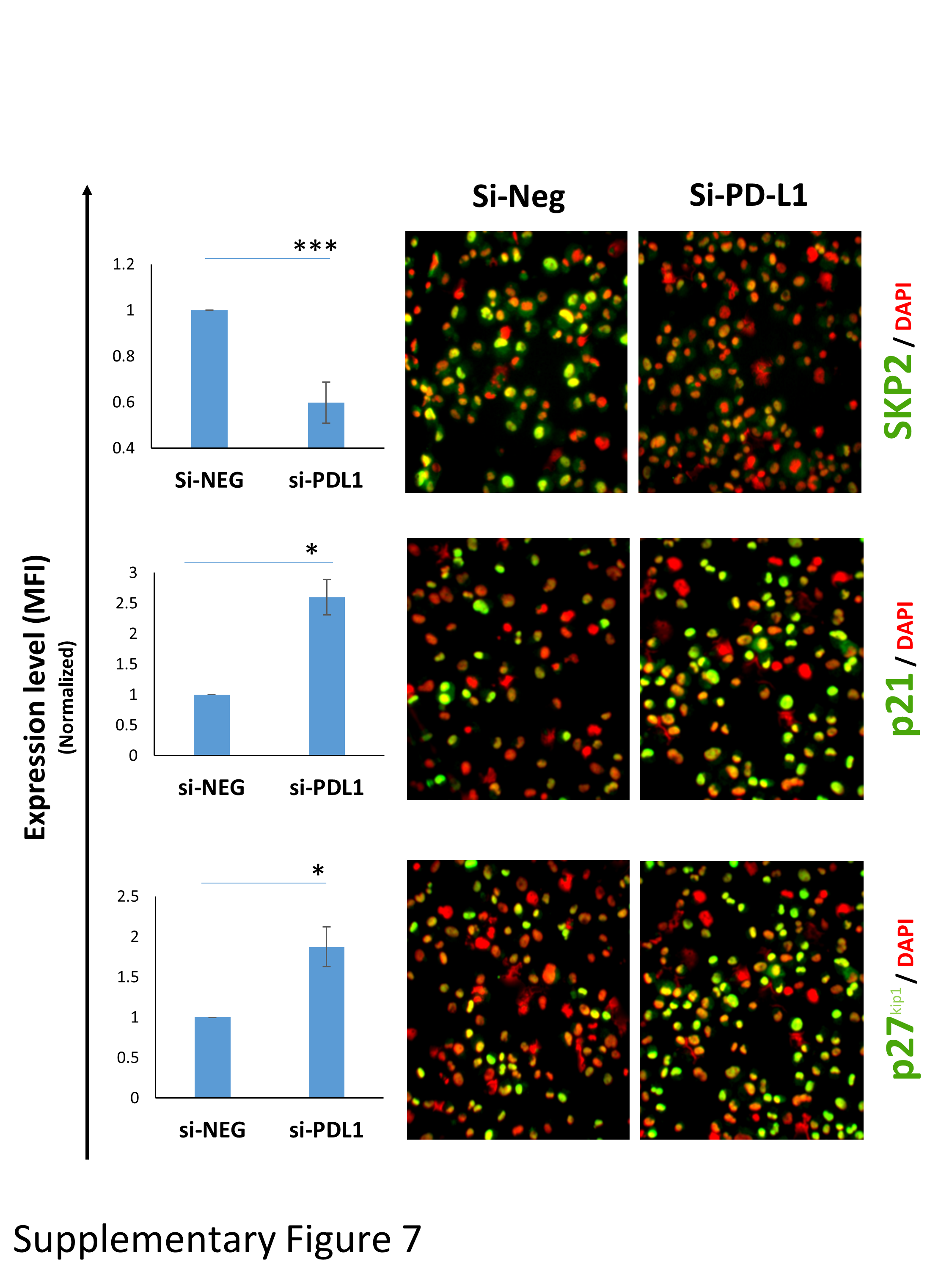

Supplement: Supplementary file 7 — Supplementary Figure 7. Left) The expression level of SKP2, p21, p27 in MDA-MB-231 cells upon transient KD of PD-L1 using specific siRNA or scrambled siRNA (siNeg) as a control. qIF showing protein expression (MFI) after normalization on siNEG cells. Data are displayed as the mean ± SEM (n=4). Right) Representative IF images upon the transient KD or the si-Neg (at x200 magnification). [file 12935_2024_3354_MOESM7_ESM.tif]

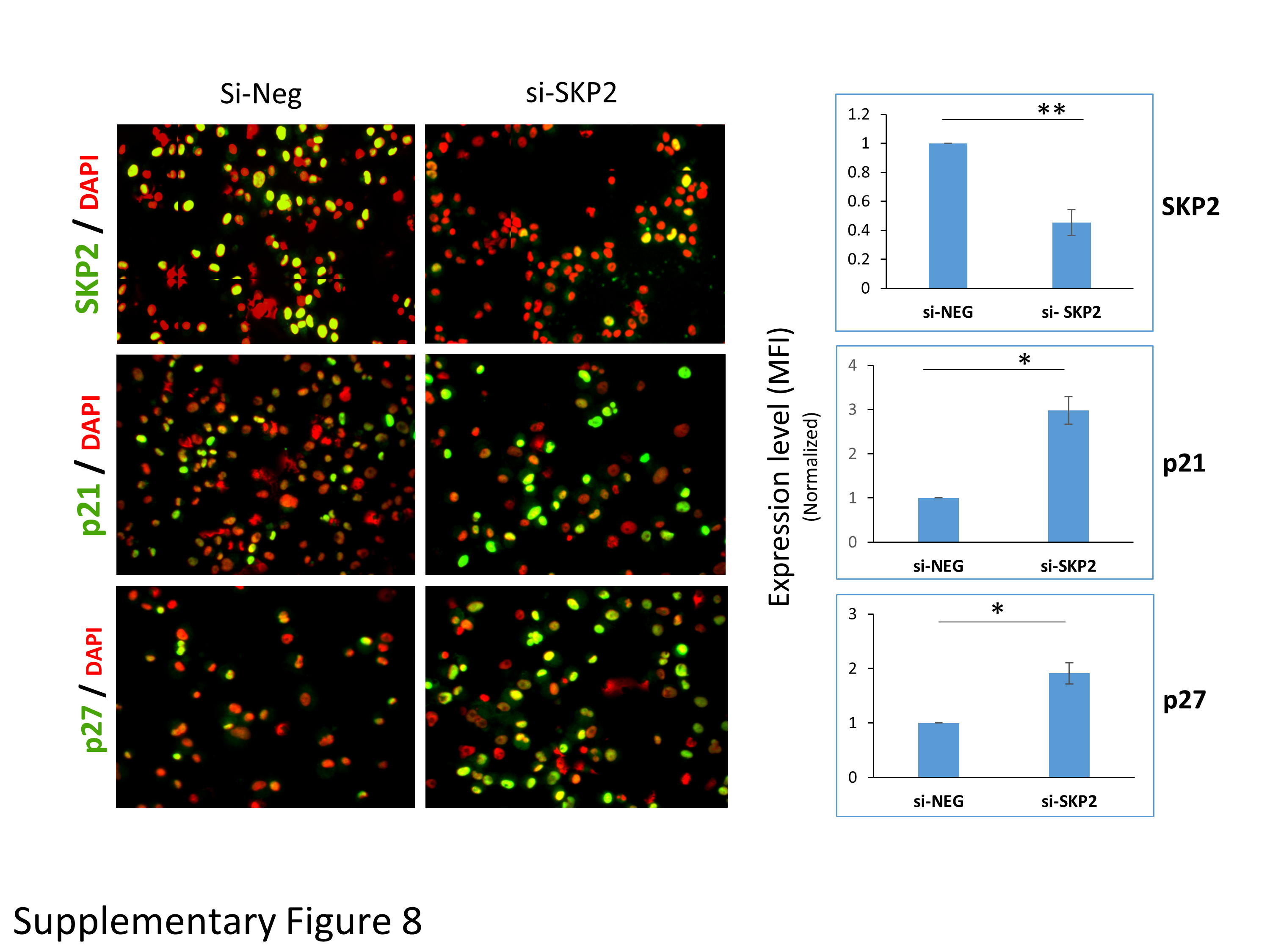

Supplement: Supplementary file 8 — Supplementary Figure 8. A) The expression level of p21 and p27 in MDA-MB-231 upon transient KD of SKP2 using si-SKP2 or scrambled siRNA (si-Neg). (Right) MFI was measured by qIF after normalization on the control (si-Neg) and displayed as a mean±SEM (n=4). (Left) Representative IF images upon transient KD using siNEG or si-SKP2 (at x200 magnification). [file 12935_2024_3354_MOESM8_ESM.tif]

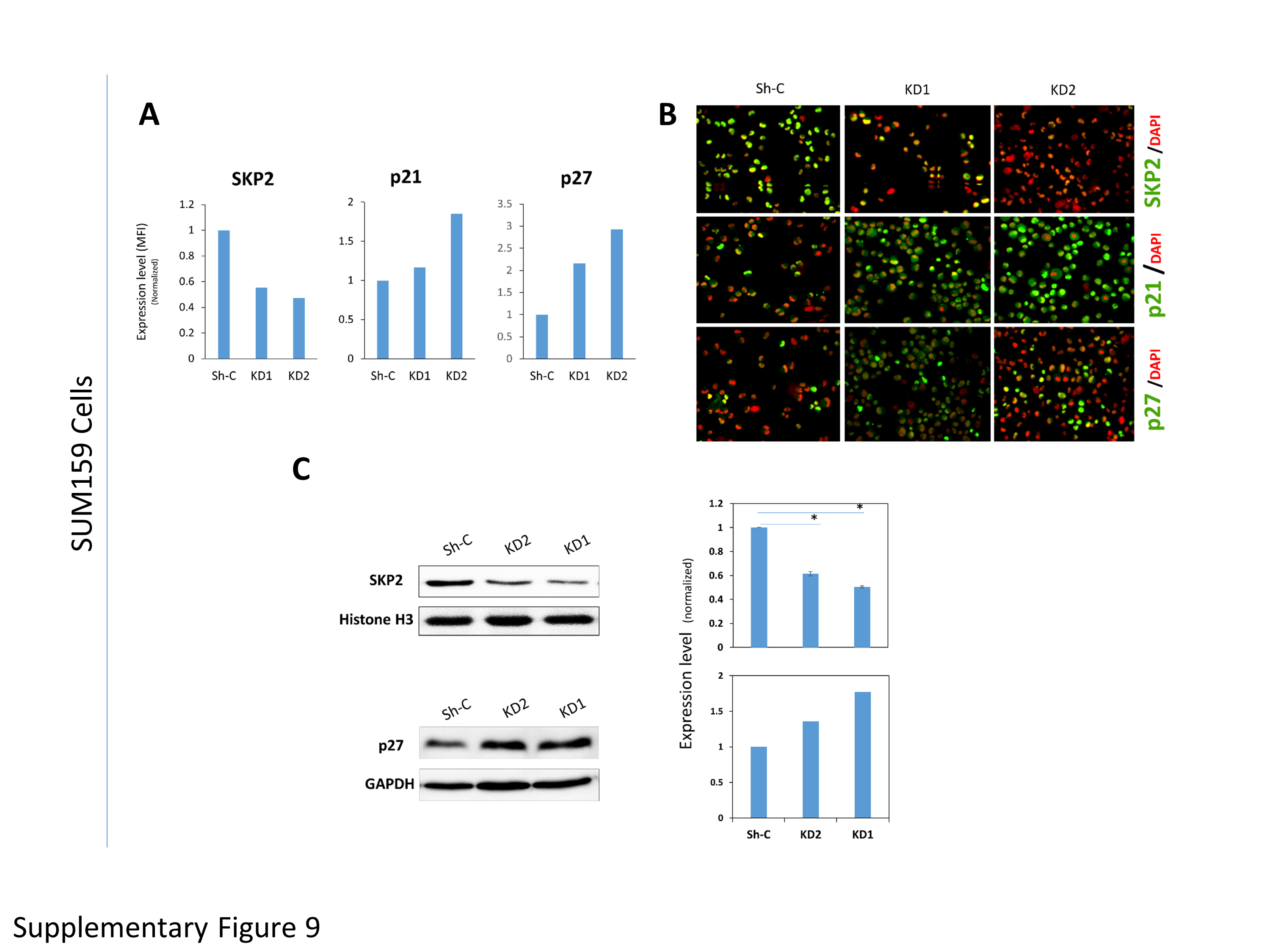

Supplement: Supplementary file 9 — Supplementary Figure 9. A) qIF showing the MFI of SKP2, p21, p27 in SUM159 PD-L1KD clones KD1 and KD2 after normalization on the control (Sh-C) (n=1). B) Representative IF images of the PD-L1KD clones and the control (at x200 magnification). C) Western blot showing SKP2 and p27 expression following PD-L1KD in SUM159 cells (left) with quantification of blots (mean±SEM, n=3) (right) for SKP2 and n=1 for p27. [file 12935_2024_3354_MOESM9_ESM.tif]
