# Supplementary material for: PD-L1 intrinsically promotes the proliferation of breast cancer cells through the SKP2-p27/p21 axis
Source: Cancer Cell Int. 2024 May 9;24:161. doi: 10.1186/s12935-024-03354-w (PMC11084005; doi:10.1186/s12935-024-03354-w)
Supplement: Supplementary file 10 — Supplementary Table 1. SiRNA sequence. [file 12935_2024_3354_MOESM10_ESM.docx]

**Supplementary Table 1. SiRNA sequence***

| **Target** | **Design/**  **Source** | **ID#** | **Sequence** | | |  |
| --- | --- | --- | --- | --- | --- | --- |
|  | | | | **Sense** | **Antisense** | |
| PD-L1 | Ambion | S26547 | | GGC AUU UGC UGA ACG CAU UTT | AAU GCG UUC AGC AAA UGC CAG | |
| SKP-2 | Inhouse | N/A | | UUA UAU AUG GAU AGU UUC Ctt | GGA AAC UAU CCA UAU AUA Att | |

*All In-house SiRNA were designed using Kay Lab siRNA/shRNA/Oligo Optimal Design website (http://web.stanford.edu/group/markkaylab/cgi-bin/) and made by Metabion International AG (Germany).
