# Supplementary material for: PD-L1 intrinsically promotes the proliferation of breast cancer cells through the SKP2-p27/p21 axis
Source: Cancer Cell Int. 2024 May 9;24:161. doi: 10.1186/s12935-024-03354-w (PMC11084005; doi:10.1186/s12935-024-03354-w)
Supplement: Supplementary file 11 — Supplementary Table 2. List of Antibodies used. [file 12935_2024_3354_MOESM11_ESM.docx]

**Supplementary Table 2. List of Antibodies used**

| **Target** | **Clone** | **Cat#** | **Company, country** | **Application** |
| --- | --- | --- | --- | --- |
| SKP2 | D3G5 | 2652 | ^1^CST | WB, IF, IHC |
| p27 | D69C12 | 3686 | CST | WB |
| p27 | F8 | sc-1641 | ^2^SCBT | IHC |
| p21 | 12D1 | 2947 | CST | WB, IF, IHC |
| Cyclin D1 | Inhouse | N/A | Abcam, UK | IF |
| FOXO3a | D19A7 | 12829 | CST | IF |

1-CST= Cell Signaling Technology, Danvers, MA, USA

2-SCBT=Santa Cruz Biotechnology, Dallas, TX, USA
