# Supplementary material for: PD-L1 intrinsically promotes the proliferation of breast cancer cells through the SKP2-p27/p21 axis
Source: Cancer Cell Int. 2024 May 9;24:161. doi: 10.1186/s12935-024-03354-w (PMC11084005; doi:10.1186/s12935-024-03354-w)
Supplement: Supplementary file 12 — Supplementary Table 3. Immunohistochemistry conditions. [file 12935_2024_3354_MOESM12_ESM.docx]

**Supplementary Table 3. Immunohistochemistry conditions**

| **Target** | **Antigen retrieval** | | **Primary Antibody** | | **Secondary Antibody (company)** |
| --- | --- | --- | --- | --- | --- |
|  | ^1^Temp | Solution | Company/  Clone/Cat# | Dilution/ Concentration |  |
| SKP2 | 121 C, 6 min | Special Citrate (Agilent Dako #S1699) | ^4^CST (D3G5) #2652 | 1:600 (0.5 µg /mL) | ^6^Biotinylated anti-rabbit (JIR), ^7^Avidin HRP (ebioscience) |
| p21 | 121 C, 6 min | ^2^Citrate, pH 6.0  (In-house made) | CST (12D1) #2947 | 1:200 (1.2 µg /mL) | Envision (Agilent Dako) |
| p27 | 121 C, 6 min | ^3^CC2 | ^5^SCBT (F-8)  #sc-1641 | 1:120 (1.7 µg /mL) | ^6^Biotinylated anti-rabbit (JIR), ^7^Avidin HRP (ebioscience) |

1. **Temp= Antigen retrieval Temperature**
2. **Citrate solution prepared In-house**
3. **CC2= Citrate based antigen retrieval solution from Ventana Medical Systems**
4. **CST= Cell Signaling Technology,**
5. **SCBT=Santa Cruz Biotechnology,**
6. **Biotinylated Goat anti-Rabbit antibody (Jackson ImmunoResearch
   Cat# 111-066-144, Ely, United Kingdom) diluted 1:1000 in 1% BSA in PBS**
7. **Avidin HRP (Cat# 18-4200-93, ebioscience, USA, diluted 1:1500) in 1% BSA in PBS**

Sections from FFPE blocks of MDA-MB-231 were used as a positive control for SKP2, while MCF-7 was a positive control for p21 and p27 staining.
